# *Campylobacter jejuni *induces transcytosis of commensal bacteria across the intestinal epithelium through M-like cells

**DOI:** 10.1186/1757-4749-2-14

**Published:** 2010-11-01

**Authors:** Lisa D Kalischuk, Frances Leggett, G Douglas Inglis

**Affiliations:** 1 Agriculture and Agri-Food Canada, 5403 1st Avenue South, T1J 4B1, Lethbridge, AB, Canada

## Abstract

**Background:**

Recent epidemiological analyses have implicated acute *Campylobacter *enteritis as a factor that may incite or exacerbate inflammatory bowel disease (IBD) in susceptible individuals. We have demonstrated previously that *C. jejuni *disrupts the intestinal barrier function by rapidly inducing epithelial translocation of non-invasive commensal bacteria via a transcellular lipid raft-mediated mechanism ('transcytosis'). To further characterize this mechanism, the aim of this current study was to elucidate whether *C. jejuni *utilizes M cells to facilitate transcytosis of commensal intestinal bacteria.

**Results:**

*C. jejuni *induced translocation of non-invasive *E. coli *across confluent Caco-2 epithelial monolayers in the absence of disrupted transepithelial electrical resistance or increased permeability to a 3 kDa dextran probe. *C. jejuni*-infected monolayers displayed increased numbers of cells expressing the M cell-specific marker, galectin-9, reduced numbers of enterocytes that stained with the absorptive enterocyte marker, *Ulex europaeus *agglutinin-1, and reduced activities of enzymes typically associated with absorptive enterocytes (namely alkaline phosphatase, lactase, and sucrase). Furthermore, in *Campylobacter*-infected monolayers, *E. coli *were observed to be internalized specifically within epithelial cells displaying M-like cell characteristics.

**Conclusion:**

These data indicate that *C. jejuni *may utilize M cells to promote transcytosis of non-invasive bacteria across the intact intestinal epithelial barrier. This mechanism may contribute to the inflammatory immune responses against commensal intestinal bacteria commonly observed in IBD patients.

## Background

Inflammatory bowel diseases (IBD) are chronic T cell-mediated diseases that are thought to result from the loss of immunologic tolerance towards commensal intestinal microorganisms [1]. Intestinal epithelial barrier dysfunction is proposed to be a primary factor contributing to IBD pathogenesis [2,3]. By facilitating the translocation of commensal bacteria across the intestinal barrier, dysfunction of the epithelium may enable the inappropriate activation of T lymphocytes that recognize and respond to constituents of the microbiota. Studies have shown that IBD patients exhibit increased rates of systemic endotoxemia [4], have higher amounts of bacterial DNA in their serum [5], and have exaggerated humoral immune responses to intestinal bacterial antigens [6-8], implying that bacterial antigens are able to translocate across the intestinal epithelium. Although several studies have observed elevated intestinal permeability ("leaky gut") and transcellular uptake of intestinal antigens in IBD patients [9-11], the mechanisms contributing to this barrier dysfunction have yet to be fully elucidated.

An increasing number of clinical studies have indicated that in some patients, IBD onset or reactivation occurs following a bout of acute bacterial enteritis [12-15]. Recently, two controlled cohort studies have implicated that enteritis incited by *Campylobacter *or *Salmonella*, which are the leading causes of bacterial enteritis in many countries, is a risk factor for subsequent development of IBD [16,17]. Although the mechanisms involved are unknown, pathogen-mediated intestinal epithelial barrier dysfunction may facilitate the translocation of commensal bacteria across the epithelium. In this regard, we have recently shown that *C. jejuni *induces translocation of non-invasive commensal bacteria across the intestinal epithelium via an uncharacterized transcellular mechanism involving lipid raft-mediated endocytosis [18,19].

Microfold or M cells are specialized epithelial cells that sample antigens from the mucosal surface and transport them via a transcellular route to the basolateral membrane (herein defined as "transcytosis"). Intestinal M cells are primary found within the follicle-associated epithelium (FAE) of Peyer's patches and isolated lymphoid follicles, but are also occasionally found interspersed amongst the absorptive enterocytes of villar epithelium [20]. While M cells are principally involved in immune surveillance of intestinal antigens, they also represent an important portal by which enteric bacteria can translocate across the intestinal barrier [21]. Studies have suggested that pathogens may exploit M cell function and upregulate the rate of bacterial transcytosis by either stimulating *de novo *formation of M cells [20,22,23] or increasing the rate of uptake for pre-existing M cells [24]. While controversy exists with respect to which of these mechanisms is responsible for increased bacterial trafficking, it is clear that certain pathogenic microorganisms can alter M cell function and increase the rate of transcytosis.

M cell trafficking of bacteria has been studied *in vitro *using polarized Caco-2 monolayers which have been stimulated to differentiate into M-like cells by co-culture with B lymphocytes [25]. Subsequent studies have shown that M-like cells capable of transporting non-invasive *Vibrio cholerae *are naturally present in Caco-2 monolayers despite the absence of B lymphocytes [26]. The resultant M-like cells display many of the *in vivo *characteristics of M cells including increased ability to ingest and transport exogenous particles, disorganized microvilli structure, decreased absorptive intestinal epithelial enzyme activities, reduced binding of the lectin, *Ulex europaeus *agglutinin-1 (UEA-1), and increased expression of the M-cell specific marker, galectin-9 [27]. We utilized Caco-2 monolayers to test the hypothesis that *C. jejuni *induces trancytosis of non-invasive commensal bacteria across the intestinal epithelium through M-like cells. Specifically, the objectives were to determine if *C. jejuni*: (1) increases transcytosis of non-invasive *E. coli *across polarized Caco-2 monolayers; (2) increases the abundance of M-like cells within Caco-2 monolayers; and (3) induces internalization of non-invasive *E. coli *specifically within M-like cells.

## Results and discussion

Our previous findings indicate that *C*. *jejuni *81-176 induces transcytosis of non-invasive *E. coli *("commensal bacteria") across polarized human colonic T84 monolayers [18]. Since other studies have shown that certain bacteria can rapidly up-regulate M cell-mediated antigen transcytosis [20,22-24,28], we examined whether *C. jejuni *utilizes M-like cells to facilitate intestinal epithelial transcytosis of non-invasive bacteria. The T84 cell line has not been characterized with respect to the presence of M-like cells; thus for this current study, we used Caco-2 monolayers in which M-like cells have previously been characterized [25,26] in order to assess whether *C. jejuni *81-176 induces translocation of non-invasive *E. coli*. In agreement with our previous study using T84 monolayers, we observed that *C. jejuni *induced internalization (~1.4-fold; *P *= 0.023) and translocation (~7.5-fold; *P *= 0.023) of *E. coli *across polarized Caco-2 monolayers. Furthermore, there were no changes in paracellular permeability to a 3 kDa dextran probe (*P *= 0.13) and transepithelial electrical resistance (TER) remained above 250 Ω × cm_2 _(*P *= 0.39; Table 1) which is indicative of intact tight epithelial junctions in this cell type. This supports our previous observations that a transcellular mechanism is responsible for *C. jejuni*-mediated *E. coli *translocation. Similarly, a recent study showed that the enteric pathogen, *Yersinia pseudotuberculosis*, also induces translocation of exogenous particles across both Caco-2 monolayers and human intestinal epithelium by an as yet uncharacterized transcellular mechanism [29]. This novel transcellular mechanism of barrier dysfunction contrasts with the paracellular mechanism described for many enteric pathogens such as enterohemorrhagic *E. coli *and *Salmonella*, which increase intestinal permeability by disrupting epithelial tight junctions [30].

**Table 1:** *E. coli *translocation and internalization, and epithelial permeability in Caco-2 monolayers treated with *E. coli *C25 alone (control) versus monolayers inoculated with *E. coli *C25 and *C. jejuni *81-176

	Control	*C. jejuni*	*P*
Translocated *E. coli *(log_10 _CFU/mL)	0.19 ± 0.19	1.42 ± 0.296	0.023
Internalized *E. coli *(log_10 _CFU/mL)	3.66 ± 0.36	4.97 ± 0.07	0.023
Initial TER (Ω × cm^2^)	298 ± 4.2	311 ± 9.4	0.28
Final TER (Ω × cm^2^)	354 ± 19.7	335 ± 2.1	0.39
Permeability (% apical dextran recovered)	0.95 ± 0.02	1.01 ± 0.02	0.13

Data are expressed as mean ± SEM, n = 3 independent experiments

*C. jejuni*-treated monolayers displayed an overall reduction in absorptive epithelial enzymatic activities (Table 2). Specifically, alkaline phosphatase was decreased by ~9.2% (*P *= 0.01), lactase was decreased by ~8.3% (*P *= 0.001), and sucrase was decreased by 28% (*P *= 0.046). This is consistent with the previously reported decrease in intestinal sucrase-isomaltase activity that occurs in the Caco-2 monolayers upon differentiation to M-like cells [25]. Notably, intestinal alkaline phosphatase has been recently show to detoxify lipopolysaccharide (a component of Gram negative bacteria cell walls) and prevent bacterial translocation [31], suggesting that reduced alkaline phosphatase activity may be one of the factors contributing to transcytosis of *E. coli *in *C. jejuni*-treated monolayers. Also, transient lactose malabsorption has been observed following campylobacteriosis [32], this may warrant further investigation.

**Table 2:** Intestinal epithelial enzyme activities for Caco-2 monolayers treated with *E. coli *C25 alone (control) verses monolayer treated with *E. coli *C25 and *C. jejuni *81-176

	Control	*C. jejuni*	*P*
	(units/g protein)	(units/g protein)	
Alkaline Phosphatase	67.6 ± 1.3	61.4 ± 0.5	0.01
Lactase	217.6 ± 1.8	199.6 ± 1.3	0.001
Sucrase	77.5 ± 4.5	55.8 ± 6.1	0.046

Data are expressed as mean ± SEM, n = 3 independent experiments

**Figure 1:**
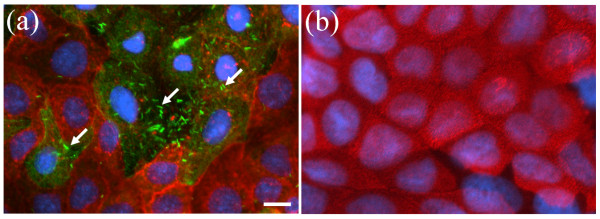
**Representative epifluorescent micrographs of Caco-2 monolayers stained with the absorptive enterocyte marker *Ulex europaeus *agglutinin-1 (red), the M-like cell specific marker galectin-9 (green diffuse cellular staining), and Hoechst nuclear stain (blue)**. (a) Monolayers treated with GFP *Escherichia coli *C25 (arrows) and *C. jejuni *81-176. (b) Monolayers treated with only GFP *E. coli *C25 (control). Bar = 2 μm.

**Figure 2:**
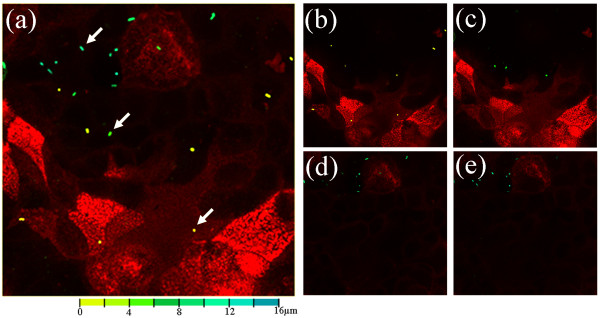
**Confocal laser sections of Caco-2 monolayers treated with *C*. *jejuni *81-176 plus non-invasive GFP *Escherichia coli *C25 (arrows) and stained with the absorptive enterocyte marker *Ulex europaeus *agglutinin-1 (red)**. *E. coli *are color-coded according to their respective depth within the monolayer from extracellular (yellow) to intracellular (blue). (a) Merge of all confocal laser sections. (b-e) Representative confocal laser sections starting from the apical surface of the monolayer (*E. coli *color-coded yellow) to the basolateral surface of the monolayer (*E. coli *color-coded blue).

Microscopic analysis revelled an increase (*P *= 0.002) in the number of cells that stained positive for galectin-9 in *C. jejuni*-treated (11.6 ± 0.5%) versus control (2.9 ± 0.5%) monolayers. Furthermore, in *C. jejuni*-treated monolayers, *E. coli *were primarily associated with Caco-2 cells displaying characteristics of M-like cells, namely those that displayed reduced apical binding of UEA-1 lectin and increased expression of galectin-9 (Figure 1). In contrast, control monolayers exhibited very few galectin-9 positive cells or associated *E. coli*. Caco-2 monolayers were sectioned using confocal laser analysis, and for each section in the z-axis, *E. coli *were coloured according to depth from yellow (extracellular) to blue (intracellular). In *C. jejuni*-treated monolayers, intracellular *E. coli *were associated with UEA-1-negative Caco-2 cells (Figure 2). The few extracellular bacteria were associated with UEA-1-positive Caco-2 cells (i.e., absorptive enterocytes). In control monolayers, only the occasional extracellular *E. coli *was observed (not shown). While cautious interpretation of data resulting from M cell staining is necessary owing to lack of a universally accepted human M cell marker, it appears that our collective data indicate that *C. jejuni *converts Caco-2 cells into cells displaying biochemical, functional, and histological features of M cells. In a similar manner, the differentiation of M-like cells has been shown to occur in response to bacterial infection and inflammation [20,22,33-35].

An important observation is that the epithelial responses appear to be specific to *C. jejuni*, since *E. coli *alone does not cause appreciable formation of M-like cells within Caco-2 monolayers. Although we are unsure of the exact mechanism, current studies have demonstrated that metabolic stress increases internalization and translocation of non-pathogenic *E.coli *across the intestinal epithelium [36,37]. We and other have previously noted swollen mitochondria (indicative of metabolic stress) in cell culture and animal models of campylobacteriosis [38-42]. It appears that mucosal pathogens often target the mitochondria as part of their common pathogenic strategy [43]. Thus, one possible explanation for our observations could be that increased M cell formation and subsequent transcytosis occur as a consequence of metabolic stress associated with *C. jejuni *pathogenesis.

Controversy continues on whether M cells arise from a distinct pre-determined lineage of crypt stem cells, or whether absorptive enterocytes exhibit phenotypic plasticity and can be "converted" to M cells [21,24]. Since immortalized Caco-2 cells are not terminally differentiated but rather resemble crypt stem cells [44,45], they are thus theoretically able to differentiate into different epithelial lineages such as absorptive enterocytes or M-like cells. The rapid timing with which *E. coli *translocation occurred following *C. jejuni *infection (i.e., 6 h), suggests that *C. jejuni *may target progenitor M-like cells within the monolayer rather than convert fully differentiated epithelial cells into M-like cells. Future studies are necessary to understand the precise mechanisms by which M-like cells originate in response to *C. jejuni*.

## Conclusion

Our data indicates that *C. jejuni *may utilize M cells to promote transcytosis of non-invasive bacteria across the intact intestinal epithelial barrier. Based on previous clinical observations, microscopic aphtoid lesions of the FAE (i.e., regions containing M cells) appear to be the initial site of inflammation in patients with Crohn's disease [46]. Notably, a recent study of patients with Crohn's disease reported increased transmucosal uptake of non-pathogenic *E. coli *across the ileal FAE despite unaltered epithelial permeability [47], suggesting that trancellular defects in the FAE contribute to the pathophysiology of IBD. M cells may play an important role in IBD pathogenesis by increasing transcytosis of commensal bacteria to the inductive sites of mucosal immune responses [48]. *Campylobacter *enteritis has been recently identified as a risk factor for IBD [16,17]. By increasing M cell-mediated trancytosis of non-invasive bacteria, *C. jejuni *may contribute to the activation of T lymphocyte-mediated immune responses against commensal bacteria commonly observed in IBD patients. Future studies will be required to determine whether *C. jejuni-*induced M cell-mediated transcytosis of commensal bacteria occurs *in vivo *and whether this causes aberrant T lymphocyte-mediated immune responses.

## Methods

### Bacteria and growth conditions

*C. jejuni *81-176, a reference clinical strain [49], was used throughout this study. For microscopy studies, *Escherichia coli *C25 transformed with GFP-expressing plasmid pMEK91 was used [50]. Inoculum was prepared by growing *C. jejuni *or *E. coli *for 14-16 hours in Columbia broth (37°C, 100 rpm, Difco, Detroit, MI) in microaerobic (10% CO_2_, 3% H_2_, 5% O_2_, balance N_2_) or aerobic atmosphere, respectively.

### Caco-2 M-like cell model

Caco-2 cells (American Type Culture Collection, Manassas, VA) were grown in Advanced Dulbecco's minimal essential medium (DMEM; Gibco Invitrogen Inc., Burlington, ON) supplemented with 10% (v/v) fetal bovine serum, 200 mM L-glutamine, 100 U/mL penicillin, 100 μg/mL streptomycin, 80 μg/mL tylosin (all from Sigma-Aldrich, Oakville, ON), and incubated at 37°C and 5% CO_2_. For *E. coli *translocation and internalization, epithelial permeability, and enzyme activity assays, cells were seeded onto Transwell filters (3 μm pore size, 1.13 cm^2^; Costar Corning Inc., Corning, NY) at 1.5 × 10^5 ^cells per filter and grown for 21 days. For microscopy, cells were seeded into chamber slides (Nalgene Nunc International, Naperville, IL) at 3 × 10^5 ^cells per well.

### E. coli *translocation and internalization assays*

*E. coli *translocation and internalization were determined as previously described [18]. Briefly, transwell-grown monolayers were washed with Hank's buffered saline (HBS; Gibco) and antibiotic-free DMEM was added to the apical and basal compartments. *E. coli *inoculum was added to the apical compartment of all monolayers to achieve a multiplicity of infection (MOI) of 100 CFU per enterocyte. Monolayers were then divided in to two groups and half were inoculated with *C. jejuni *at a MOI of 100, whereas the other half received an equivalent volume of sterile broth (control treatment).

Following incubation, *E. coli *recovered in the basal compartment (indicating translocation) were enumerated by spreading serial dilutions onto MacConkey agar, incubating the cultures aerobically at 37°C, and enumerating at the dilution yielding 30-300 colony forming units (CFU) per culture. A preliminary experiment confirmed that 6 h was the minimum optimal incubation time for assessing *E. coli *translocation. To assess *E. coli *internalization, treated monolayers were washed with HBS and incubated for 1 hour with DMEM containing gentamicin (250 μg/mL; Sigma). Monolayers were then washed, lysed with 0.1% Triton X-100 in PBS (500 μL), and viable bacteria were enumerated as described above. A preliminary experiment confirmed that *E. coli *were killed by the gentamicin treatment. Transepithelial electrical resistance was monitored at the beginning and end of each experiment with an electrovoltohmeter (World Precision Instruments, Sarasota, FL). Only monolayers with an initial TER > 225 Ω × cm^2 ^were used.

### Epithelial permeability assay

Transwell-grown monolayers were inoculated with *E. coli *± *C. jejuni *as described above, and monolayers were washed with Ringer's buffer 6 hours after inoculation. A 3 kDa FITC-dextran probe (100 mM in Ringer's buffer; 500 μL per well; Molecular Probes, Eugene, OR) was added to the apical compartment and 1 mL of Ringer's buffer was added to the basal compartment, and the monolayers were incubated for 3 hours at 37°C as described previously [18]. Samples (200 μl) were collected from the basal compartment and the absorbance at 485 nm was measured. Data were expressed as % apical dextran recovered in the basal compartment.

### Epithelial enzyme activity assays

Transwell-grown monolayers were inoculated with *E. coli *± *C. jejuni *as described above. After 6 hours, monolayers were washed with PBS and lysed with 0.2% Triton X-100 in PBS (500 μL per well) on ice for 20 minutes. Cell lysates were clarified by centrifugation (2 minutes, 16,000 (g). Total protein concentration of the lysates was determined using the Bradford protein assay (Bio-Rad, Mississauga, ON) and were calculated by interpolation of a standard curve generated with known concentrations of bovine serum albumin (BSA).

Alkaline phosphatase activity was measured by hydrolysis of p-nitrophenyl phosphate (pNPP; Sigma) reagent according to the manufacturer's instructions. Briefly, cell lysates (20 μL) were incubated for 30 minutes at 37°C with pNPP reagent (500 μL). The reaction was stopped with 2 M NaOH (150 μL) and measured spectrophotometrically at A_410 _nm. Enzyme activity was calculated by interpolation of a standard curve generated with alkaline phophatase of defined activity. Enzyme activity was expressed as units/g protein, where 1 unit is defined as the amount of enzyme that hydrolyzed 1 μM of pNPP per minute at 37°C.

Sucrase and lactase activities were measured according to the method of Dahlqvist [51]. In brief, cell lysates (50 μL) were incubated with sucrose or lactose (50 μL of 100 mM disaccharide in 0.1 M maleate buffer, pH 6; Sigma) in each of two separate tubes. One set of tubes, used to measure background endogenous glucose in the sample, was heated at 95°C for 2 minutes. The second set of tubes was placed in a 37°C water bath for 4 hours followed by 95°C for 2 minutes. Glucose oxidase reagent (200 μL; Sigma) was added to each tube and then incubated at 37°C for 1 hour. A coloured product of O-dianisidine, which is produced based on the amount of glucose liberated in the samples, was measured spectrophotometrically at A_420 _nm. Glucose in each lysate was calculated by interpolation of a glucose standard curve. Enzyme activity was expressed as units/g protein, where 1 unit is defined as the amount of enzyme that liberated 1 μM of glucose in 1 hour.

### M cell staining, epifluorescent and confocal microscopy

Confluent Caco-2 monolayers grown on chamber slides were inoculated with *E. coli *± *C. jejuni *as described above. Caco-2 monolayers were washed with PBS 6 hours after inoculation, and fixed in paraformaldehyde (2%). Galectin-9 staining was performed as previously described [27]. Briefly, slides were washed with PBS, incubated with glycine (1% in PBS) for 15 minutes, and washed with PBS. Cells were permeabilized for 10 minutes with Triton X-100 (0.5% in PBS), blocked with BSA (2% in PBS), incubated with goat anti-human Galectin-9 antibodies (1% in PBS; R&D Systems Inc., Minneapolis, MN) followed by Alexa-488 conjugated anti-goat IgG (0.2% in PBS; Molecular Probes). Monolayers were then washed with PBS and incubated for 30 minutes with TRITC-conjugated UEA-1 (5 μg/mL in PBS; Sigma). After washing with PBS, slides were stained for 15 minutes with Hoechst nuclear stain (1 μM; Molecular Probes) and washed with PBS. Coverslips were mounted with Aqua-Mount (Lerner Laboratories, Pittsburgh, PA) according to the manufacturer's instructions. Slides were examined by epifluorescent microscopy using appropriate filters. Slides were blinded by taping the labels, and a grid was drawn on the back of each slide. The same section of the grid was examined by epifluorescent microscopy and scored for total number of cells and number of M cells (i.e., galectin-9 positive, UEA negative). Confocal optical sectioning was carried out using an inverted microscope equipped with a confocal laser scanning imaging system (Leica Microsystems, Wetzler, Germany).

### Statistical analysis

Each assay was conducted at least three times on separate occasions. For each replicate, observations were conducted at least in triplicate, and mean values were used for analysis. Data are expressed as means ± SEM. Unpaired Student's t-test were used to compare means of control versus *C. jejuni*-treated samples (GraphPad InStat software, GraphPad Software Inc., San Diego, CA). *P *≤ 0.05 was considered significant.

## Competing interests

The authors declare that they have no competing interests.

## Authors' contributions

LKT participated in the design of the study, performed experiments, conducted data analysis, and drafted the manuscript. FL conducted confocal microscopy and image analysis. GDI participated in the design of the study and edited the manuscript. All authors read and approved the final manuscript.
